# Systematic review and meta-analysis of prognostic microRNA biomarkers for survival outcome in laryngeal squamous cell cancer

**DOI:** 10.1186/s12935-021-02021-8

**Published:** 2021-06-22

**Authors:** Yan Huang, Min Gu, Yiting Tang, Zhiqiang Sun, Judong Luo, Zhe Li

**Affiliations:** 1 Department of Radiotherapy, The Affiliated Changzhou No.2 People’s Hospital of Nanjing Medical University, Changzhou, China; 2 Department of Head and Neck Surgery, Graduate School of Dalian Medical University, Dalian, China; 3 Department of Stomatology, Affiliated Third Hospital of Soochow University, The First People’s Hospital of Changzhou City, Changzhou, China; 4 State Key Laboratory of Radiation Medicine and Protection, School of Radiation Medicine and Protection, Soochow University, Suzhou, China; 5 Department of Breast Surgery, Shuguang Hospital Affiliated to Shanghai University of Traditional Chinese Medicine, Shanghai, China

**Keywords:** Laryngeal cancer, Overall survival, Disease-free survival, Prognosis, MicroRNAs

## Abstract

**Background:**

Laryngeal carcinoma is a primary malignant tumor originating from the laryngeal mucosa, and its pathogenesis is not fully understood. It is a rare type of cancer that shows a downward trend in the 5-year survival rate. In clinical practice, dysregulated microRNAs are often observed in patients with laryngeal cancer. In recent years, an increasing number of studies have confirmed that the strong biomarker potential of microRNAs. We conducted a systematic review and meta-analysis to identify and highlight multiple microRNAs as biomarkers for disease prognosis in patients with laryngeal cancer.

**Methods:**

We actively searched the systematic reviews in PubMed, Embase, Web of Science and The Cochrane Library to select the studies that met the proposed guidelines. A total of 5307 patients with laryngeal cancer were included in this study to evaluate the association between microRNAs expression levels and patient outcomes. For overall survival in the clinical stage, a hazard ratio (HR) and corresponding 95% confidence interval (CI) are calculated to assess the effect of survival.

**Results:**

A total of 36 studies on microRNAs and laryngeal cancer recovery were included in this meta-analysis. The selected endpoints for these studies included overall survival (OS) and disease-free survival (DFS).The comorbidities of overexpression and underexpression of microRNAs were 1.13 (95% CI 1.06–1.20, P < 0.05) and 1.10 (95% CI 1.00–1.20, P < 0.05), respectively.

**Conclusion:**

MiRNA-100, miRNA-155, miRNA-21, miRNA-34a, miRNA-195 and miR-let-7 are expected to be potential noninvasive and simple markers for laryngeal cancer.

## Introduction

Laryngeal cancer is the second most common head and neck cancer (HNC), accounting for 1 to 5% of systemic malignancies. A recent study showed that the 5-year overall survival rate for all HNC sites was 51.4:50.3% for the oral cavity, 41.1% for the oropharynx, 35.0% for the hypopharynx and 63.9% for the larynx [1]. Fortunately, in the last decade, there have been major advances in the treatment of throat cancer [2]. Tobacco and alcohol are closely related factors of laryngeal cancer and lung cancer. These cancer types are more common among men because they are more exposed to these factors. Although tumors can develop anywhere in the throat, the glottis is the most common site, followed by the supraglottis and the subglottis [3]. Therefore, there is an urgent need to identify biomarkers for HNC to facilitate accurate diagnoses, provide information for patients and guide treatment.

MicroRNAs are small endogenous RNA molecules containing approximately 22 nucleotides that regulate gene expression by binding to specific messenger RNAs [4]. MicroRNAs are often located in genomic regions associated with cancer and are involved in the regulation of gene function [5]. It is believed that microRNAs can be used as tumor suppressor genes as well as oncogenes, and play an important role in the occurrence and development of cancer [6, 7]. MicroRNAs are short-stranded noncoding RNAs that play a very important role in malignant tumorigenesis, and they regulate the expression of tumor-related genes at the posttranscriptional level through specific binding to their target genes [8, 9]. MicroRNAs are highly stable and can be measured in biological fluids, including serum, plasma, and saliva, allowing for quick results and repeated analysis during and after treatment. This property strengthens the role of microRNAs as biomarkers and their great potential in the field of oncology [10]. MicroRNAs have been widely researched, and the results indicate that they may provide a sensitive method for the detection, monitoring and prognosis of laryngeal squamous cell carcinoma [11, 12]. In this study, we conducted a systematic review of the existing literature on this topic in PubMed, Embase, Web of Science and The Cochrane Library. Then, we conducted a meta-analysis of the survival rates (including overall survival [OS] and disease-free survival [DFS]) of patients expressing different levels of microRNAs.

## Methods

### Selection criteria

This meta-analysis was conducted based on an originally conceived protocol. To be qualified, studies had to meet all the following criteria: (1) the study discussed the expression of microRNAs in patients with laryngeal squamous cell carcinoma. (2) The study investigated the relationship between microRNAs expression levels and the survival status of cancer patients. (3) The study was published in full text and reported the risk ratio (HR) of DFS or OS based on microRNA status, with a confidence interval (CI) of 95%.

Studies that met one of the following criteria were excluded: (1) case reports, conference proceedings, letters, or reviews/meta-analyses; (2) thyroid and oral tumors; (3) animal studies; (4) laboratory studies; or (5) incomplete data (no NLR HR for OS/DSS). Incomplete study data (for example, studies that included only Kaplan–Meier curves or HRs that did not report a 95% confidence interval) were not initially ruled out. When this occurred, we contacted the authors and attempted to obtain the raw data.

### Literature search strategy

A comprehensive search was performed in PubMed, EMBASE, and Web of Science databases from their inception through December 5, 2020. The search terms were “(Laryngeal Neoplasms OR Neoplasms, Laryngeal OR Laryngeal Neoplasm OR Neoplasm, Laryngeal OR Larynx Neoplasms OR Larynx Neoplasm OR Neoplasm, Larynx OR Neoplasms, Larynx OR Cancer of Larynx OR Larynx Cancers OR Laryngeal Cancer OR Cancer, Laryngeal OR Cancers, Laryngeal OR Laryngeal Cancers OR Larynx Cancer OR Cancer, Larynx OR Cancer of the Larynx) AND (MicroRNAs OR MicroRNA OR miRNAs OR Micro RNA OR miRNA OR Primary MicroRNA OR Primary miRNA OR pri-miRNA OR RNA, Small Temporal OR pre-miRNA OR pre-miRNA) AND (Prognosis OR Prognoses OR Prognostic Factors OR Factor, Prognostic OR Factors, Prognostic OR Prognostic Factor)”. In addition, we manually searched the selected review articles and reference lists of preliminary studies to ensure complete coverage.

### Data extraction

To ensure the accuracy of data extraction, two authors extracted data according to the inclusion and exclusion criteria, and a third author assisted in making the final decision on the disputed information. Data were extracted, including author name, year of publication, country, tumor type, total number of patients and test method, follow-up time, microRNAs expression, HR and 95% CI. Meta-analyses were performed as required by the item statement of the systematic review and meta-analysis priority report.

### Quality assessment

Methodological quality was systematically reviewed and meta-analyzed based on the quality assessment template of the National Heart, Lung and Blood Institute (NHLBI). Based on the opinions of the two reviewers, each study was subjectively rated as having a “high”, “moderate” or “low” risk of bias rating (Table 1).

**Table 1:** Quality assessment of the selected studies for meta-analysis

S. no	Criteria	High (0–55%)	Moderate (56–78%)	Low (79–100%)
1	Purpose of this study	36	–	–
2	Eligibility criteria	25	6	5
3	Sample size adjustment	36	–	–
4	Research group of people	36	–	–
5	Cut-off criteria (follow-up)	33	3	–
6	Range of anatomical parts	36	–	–
7	Definition of the measurement used	36	–	–
8	Outcome assessment (OS, DFS)	31	5	–
9	Outcome measures (HR, CI)	26	–	10
10	Follow-up rate	27	9	–
	Total selected studies	20	8	8

### Statistical methods

Meta-analysis was performed on data from multiple included studies. Comprehensive meta-analysis software (RevMan5.3 and Stata 15.1) was used. A combined HR > 1 indicates that the prognosis of the group with elevated microRNA expression was poor. The red square shows a combined effect estimate of survival in patients with laryngeal cancer arranged by microRNA expression. We attempted to explore the heterogeneity of the results through subgroup analysis.

### Publication bias

We used Egger’s bias assessment graph test to construct funnel plots (scatter plots constructed using standard error [Y axis] and log (HR) [X axis]) for all included studies. The symmetry of the study distribution on the regression line is inversely proportional to the size of the publication bias in the meta-analysis. In the process of data selection and quality assessment, any differences were resolved through discussion among the reviewers.

## Results

### Study selection

**Fig. 1.**
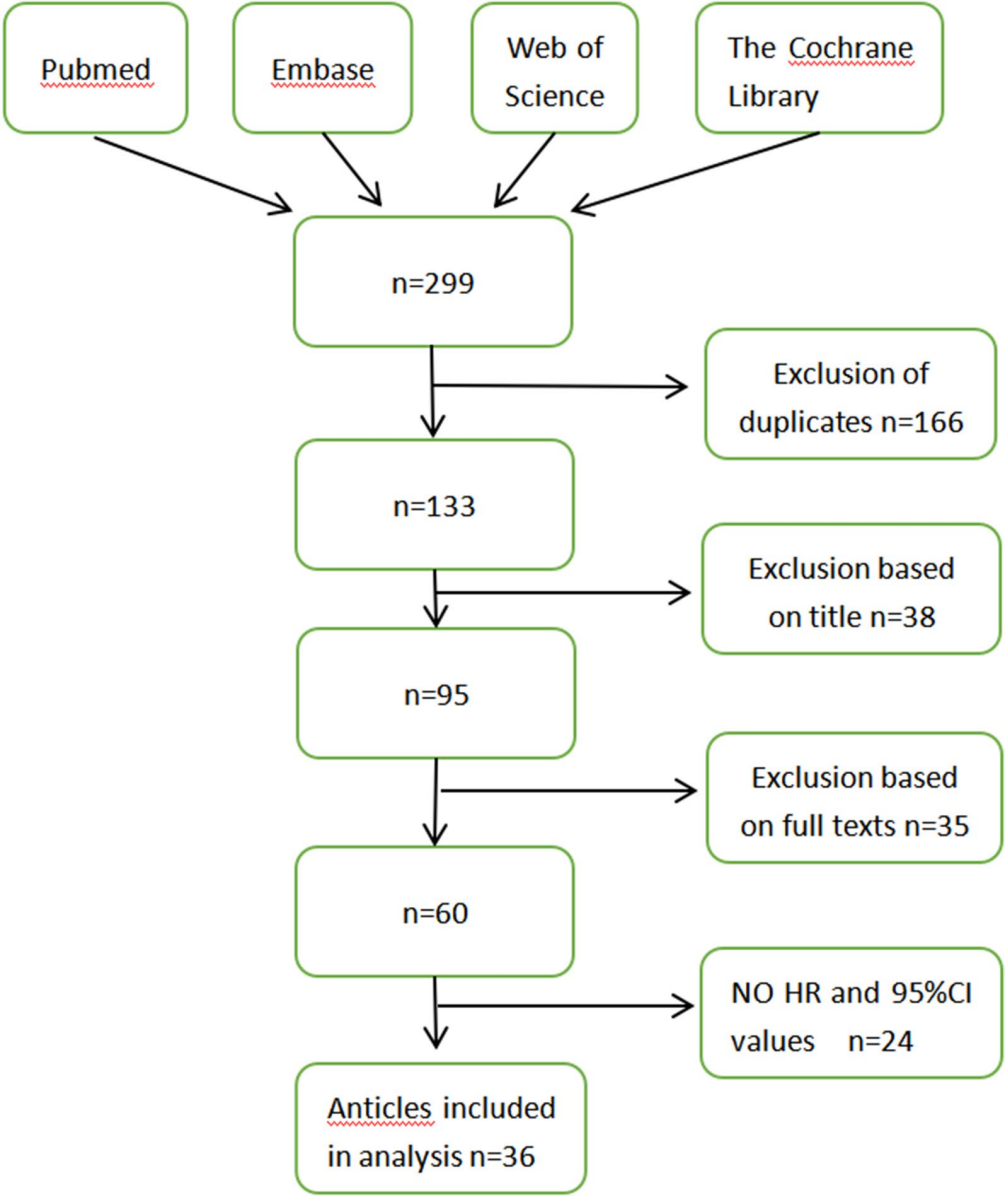
Flow chart describing search strategy

We found a total of 299 records and placed them into the EndNote Web reference manager to delete duplicate articles. A total of 36 studies were included in the meta-analysis. The details are shown in Fig. 1.

### Study characteristics

The studies included in this systematic review included a sample of 36 research articles. The average patient age in most studies was approximately 60. We found that these studies originated from eight countries around the world, including The Netherlands (n = 1), Italy (n = 2), Germany (n = 1), Brazil (n = 2), U.S. A. (n = 6), Athens (n = 1), Japan (n = 1), and China (n = 22). MicroRNA expression was found in preserved tissue samples (23 studies), serum (10 studies) and plasma (3 studies). Eight studies used TaqMan low-density arrays to detect microRNAs expression profiles, and 27 studies used RT-PCR to detect microRNAs expression. The average period for follow-up studies was 3–7 years. In all the studies included in the systematic review and meta-analysis, a total of 24 microRNAs were reported. Of the 24 microRNAs included, 10 microRNAs were upregulated, and 14 microRNAs were downregulated. Table 2 describes the research design and patient information for all 36 studies (Table 2).

**Table 2:** Characteristics of the included studies of the meta-analysis

Author	Year	MiRNA	Case	Anatomic location	Assay Method	Country	Gender	Stage	Metastasis	Age	Outcomes	miRNA dysregulation
Shuang et al. [13]	2017	miR-195	122	Glottis 61 Supraglottis 42 Subglottis 19	qRT-PCR	China	Male (65.5%)	TI + T2 30 T3 + T4 92	N0 81 N + 41	53–69 y	OS	Down regulated
Zhao et al. [14]	2018	miR-145	132	Glottic 76 Supraglottic 56	qRT-PCR	China	Male (86.3%)	T2 51 T3 + T4 81	N0 61 N+ 71	48–84 y	OS	Down regulated
Gao et al. [15]	2019	miR-145-5p	188	Glottic 101 Supraglottic 83 Subglottic 4	TCGA	China	Male (88.8%)	T1 + T2 111 T3 + T4 77	N0 142 N+ 46	≤ 60y 99 > 60y 89	OS	Down regulated
Li et al. [16]	2015	miR-101	80	Larynx	qRT-PCR	China	Male (70%)	T1 + T2 40 T3 + T4 40	N0 45 N + 35	≤ 60y 48 > 60y 32	OS	Down regulated
de Jong et al. [17]	2015	miR-203	34	NA	qRT-PCR	The Netherlands	NA	NA	NA	NA	OS	Down regulated
Ding and Qi [18]	2019	miR-195	182	Supraglottic 50 Glottic 95 Subglottic 37	qRT-PCR	China	Male (65.3%)	NA	N0 110 N+ 72	NA	OS	Down regulated
Fang et al. [19]	2019	miR-29c-3p	66	Supraglottic 19 Glottic 45 Subglottic 2	qRT-PCR	China	Male (93.9%)	T1 + T2 21 T3 + T4 45	N0 36 N+ 30	≤ 60y 26 > 60y 40	OS	Down regulated
He et al. [20]	2017	miR-300	133	Larynx	qRT-PCR	China	Male (65.4%)	T1 + T2 67 T3 + T4 66	N0 73 N+ 60	≤ 50y 47 > 50y 86	OS	Down regulated
Hui et al. [21]	2019	miR-10a miR-16–2	22	Larynx	TCGA	China	NA	NA	NA	NA	OS, RFS	Down regulated
Re et al. [22]	2015	miR-34c-5p	90	Supraglottic 19 Transglottic 66 Subglottic 5	qRT-PCR	Italy	Male (96.6%)	T3 60 T4 30	N0 29 N+ 61	Mean 66.51	OS	Down regulated
Xu et al. [23]	2016	miR-149	97	Larynx	qRT-PCR	China	NA	T1 + T2 59 T3 + T4 38	N0 69 N+ 28	≤ 60y 46 > 60y 51	OS	Down regulated
Tian et al. [24]	2014	miR-203	56	Glottic 30 Supraglottic 26	qRT-PCR	China	Male (71.43%)	T1 + T2 24 T3 + T4 32	N0 23 N+ 23	≤ 59y 32 > 59y 24	OS	Down regulated
Hess et al. [25]	2017	miR-155 miR-146a miR-200b	149	Oropharynx 77 Hypopharynx 72	qRT-PCR	Germany	NA	NA	NA	NA	OS, RFS	Down regulated
Zhao et al. [26]	2018	miR-181a	127	Supraglottic 50 Glottic 77	qRT-PCR	China	Male (88.6%)	T1 + T2 53 T3 + T4 74	N0 65 N+ 62	≤ 60y 79 > 60y 48	OS	Down regulated
Zhao et al. [27]	2018	miR-196b	113	Supraglottic 43 Glottic 70	qRT-PCR	China	Male (85%)	T1 + T2 47 T3 + T4 66	N0 65 N+ 48	≤ 60y 71 > 60y 42	OS	Upregulated
Zhao et al. [28]	2018	miR-155	120	Supraglottic 46 Glottic 74	qRT-PCR	China	Male (89.2%)	T1 + T2 67 T3 + T4 53	N0 88 N+ 32	≤ 60y 63 > 60y 57	OS	Upregulated
Guan et al. [29]	2016	miR-675	62	Larynx 46 Hypopharynx 14 Oropharynx 2	qRT-PCR	China	Male (94%)	NA	NA	Mean 64	OS	Upregulated
Arantes et al. [30]	2017	miR-21 miR-494 miR-720	71	Oropharynx 35 Larynx 28 Hypopharynx 8	TCGA	Brazil	Male (95.8%)	T1 + T2 46 T3 + T4 25	N0 35 N+ 36	NA	OS	Upregulated
Avissar et al. [31]	2009	miR-21	169	Larynx	qRT-PCR	USA	Male (68%)	T1 + T2 46 T3 + T4 118	NA	Mean 61.5	OS	Upregulated
Langevin et al. [32]	2011	miR-137	67	Larynx	qRT-PCR	USA	Male (74.6%)	T1 + T2 35 T3 + T4 32	N0 31 N+ 36	Mean 62.4	OS	Upregulated
Qiang et al. [33]	2019	miR-31	55	Larynx	qRT-PCR	China	Male (57.1%)	T1 + T2 21 T3 + T4 35	NA	Mean 63.2	OS	Upregulated
Wu et al. [34]	2014	miR-9	103	Supraglottic 66 Glottic 37	qRT-PCR	China	Male (47.5%)	T1 + T2 55 T3 + T4 48	N0 31 N+ 74	≤ 60y 41 > 60y 62	OS	Upregulated
Wu et al. [35]	2014	miR-19a	83	Supraglottic 35 Glottic 48	qRT-PCR	China	Male (68.6%)	T1 + T2 52 T3 + T4 31	N0 54 N + 29	≤ 56y 42 > 56y 41	OS	Upregulated
Zhang et al. [36]	2015	miR-23a	52	Larynx	qRT-PCR	China	Male (86.5%)	T1 + T2 24 T3 + T4 28	N0 34 N + 18	≤ 60y 22 > 60y 30	OS	Upregulated
Hu et al. [37]	2014	miR-21	46	Glottic 33 Supraglottic 11 Subglottic 2	qRT-PCR	China	Male (91.3%)	T0 + T1 + T2 21 T3 + T4 25	NA	Mean 59.2	OS	Upregulated
Saito et al. [38]	2013	miR-196a	84	Larynx	qRT-PCR	Japan	NA	NA	NA	NA	OS	Upregulated
Re and Magliulo [39]	2017	miR-34c-5p	43	Supraglottic 8 Transglottic 33 Subglottic 2	qRT-PCR	Italy	Male (97.67%)	T3 31 T4 12	N0 27 N+ 16	Mean 66.51	DFS	Downregulated
Shen et al. [40]	2012	miR-34a	69	Larynx	qRT-PCR	China	NA	T1 + T2 42 T3 + T4 27	N0 24 N+ 45	≤ 60y 33 > 60y 36	DFS	Downregulated
Danielle Maia [41]	2015	mIR-296-5P	34	Supraglottic 7 Glottic 27	TCGA	Brazil	Male (88.2%)	TI 16 T2 18	NA	≤ 60y 16 > 60y 18	DFS	Downregulated
Ogawa et al. [42]	2012	miR-34a	24	Larynx	TCGA	Japan	Male (66.6%)	T3 10 T4 14	NA	≤ 60y 10 > 60y 14	DFS	Downregulated
Pantazis et al. [43]	2020	miR-20b-5p	105	Larynx	qRT-PCR	Athens	Male (63.1%)	T1 + T2 31 T3 + T4 74	NA	NA	DFS	Downregulated
Wilkins [44]	2019	miR-100	136	Larynx	TCGA	USA	NA	NA	NA	NA	DFS	Downregulated
Li et al. [45]	2019	miR-424-5p	106	Glottic 55 Supraglottic 40 Subglottic 3 Transglottic 8	TCGA	China	Male (93.3%)	T1 + T2 58 T3 + T4 48	N0 80 N+ 26	≤ 60y 47 > 60y 59	DFS	Upregulated
Childs et al. [46]	2009	miR-let-7d	73	Oropharynx 32 Hypopharynx 9 Larynx 32	qRT-PCR	USA	Male (68%)	T1 + T2 17 T3 + T4 56	NA	≤ 60y 30 > 60y 43	OS	Downregulated
Liu et al. [47]	2017	miR-let-7a	131	Larynx	qRT-PCR	China	Male (33%)	T1 + T2 51 T3 + T4 80	NA	≤ 60y 88 > 60y 43	OS	Downregulated
Wilkins et al. [48]	2018	miR-let-7a	2083	Pharynx 1458 Larynx 625	TCGA	USA	Male (24.5%)	T1 + T2 541 T3 + T4 1542	NA	NA	OS	Downregulated

### Meta-analysis

**Fig. 2.**
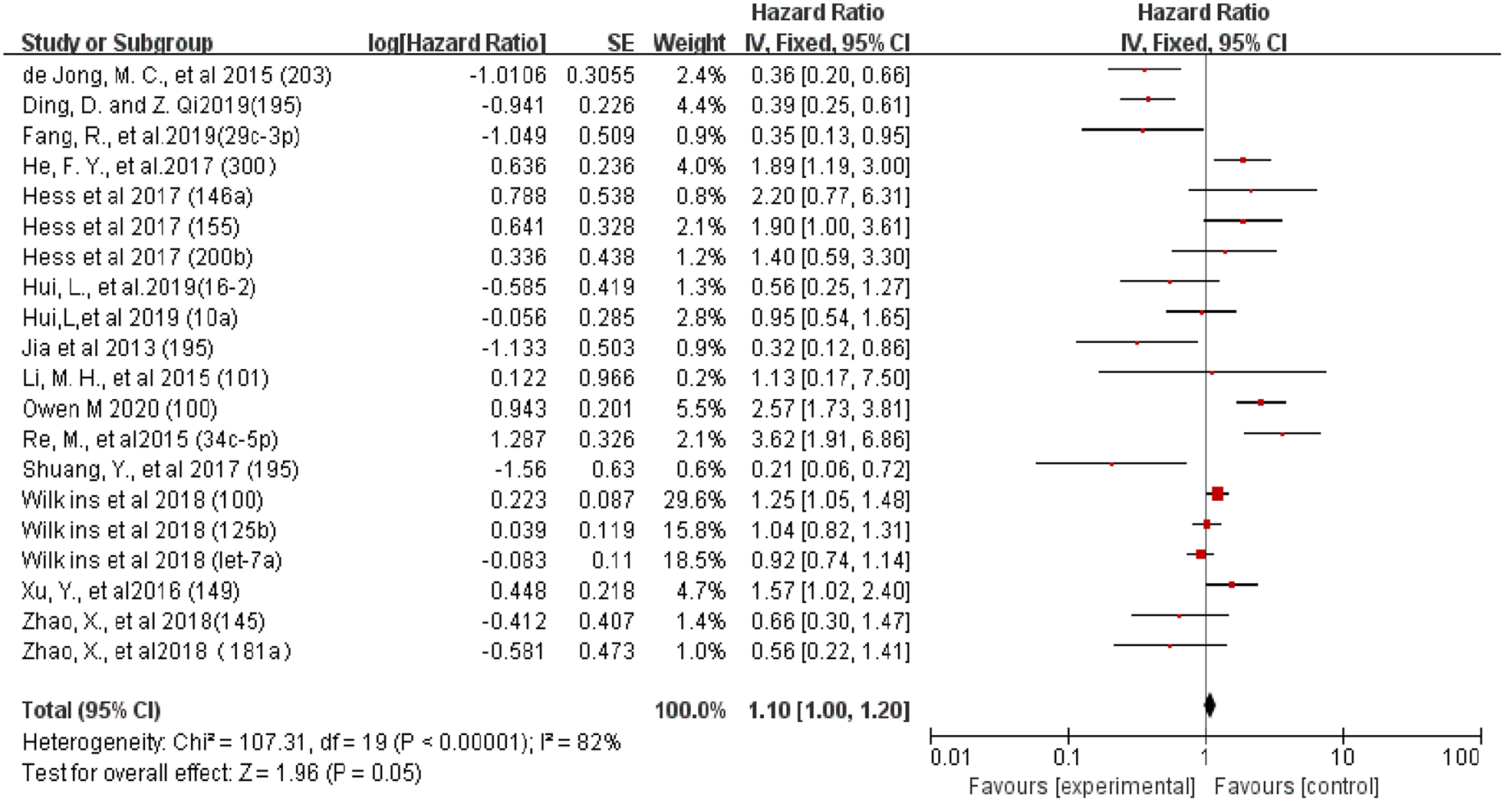
Meta-analysis of downregulated miRNA expression for overall survival in LSCC

**Fig. 3.**
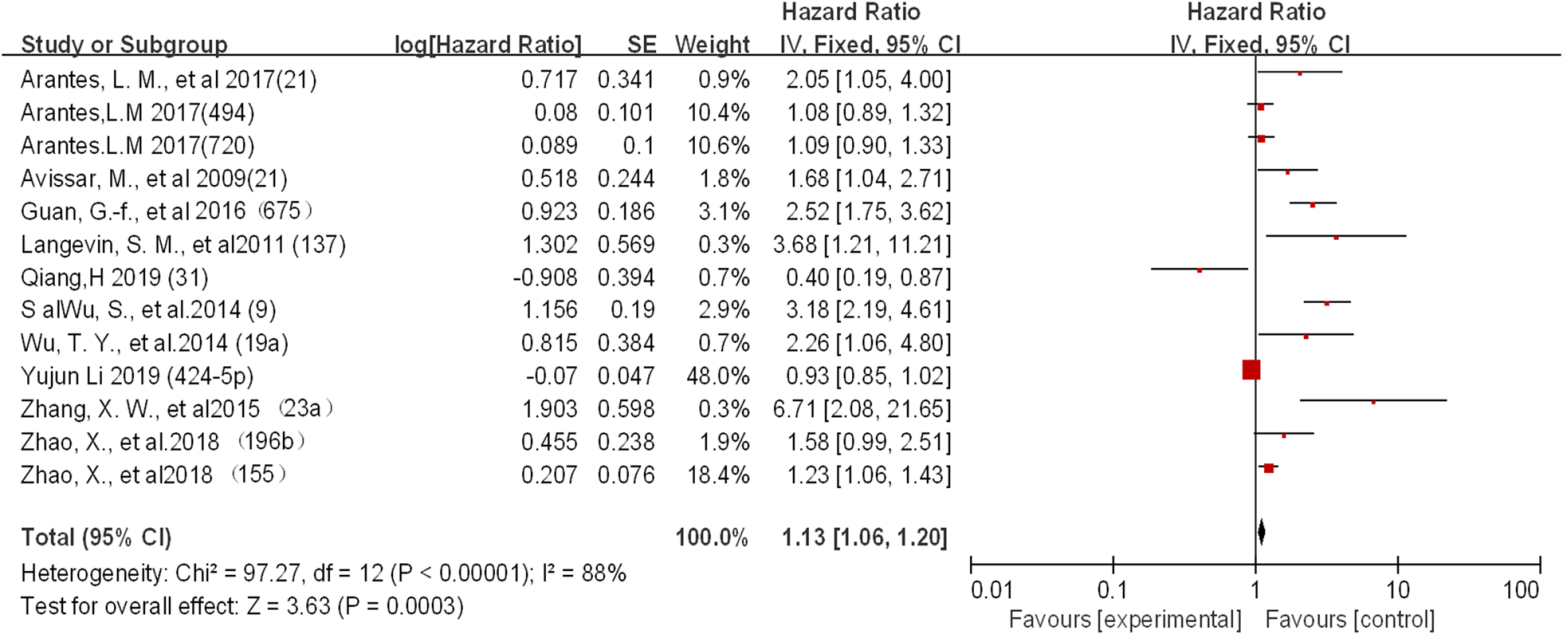
Meta-analysis of upregulated miRNA expression for OS in LSCC

**Fig. 4.**
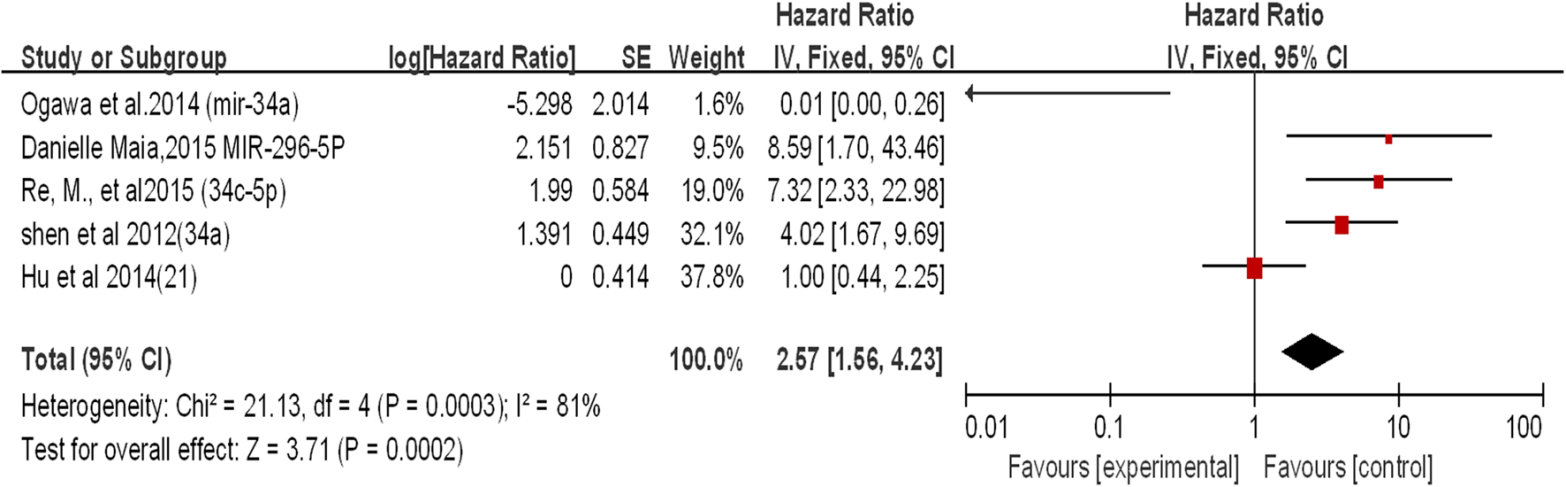
Meta-analysis of upregulated and downregulated miRNA expression for disease-free survival in LSCC

The association of OS with at least one microRNA in laryngeal squamous cell carcinoma (LSCC) was reported in 36 studies. The expression of these molecules was detected primarily at the RNA level, and the upregulated expression was compared with that in normal tissues. The meta-analysis pooled HR and 95% CI (n = 36) from studies with OS as the endpoint, providing an overall effect of a downregulated microRNA size estimate (HR) of 1.10 (95% CI 1.00–1.20), while the estimated pooling effect size (HR) of upregulated microRNAs was 1.13 (95% CI 1.06–1.20). The effect size estimate (HR) for studies using DFS as their survival endpoint was 2.57 (95% CI 1.56–4.23). When OS was used as a survival endpoint, there was no significant difference between the upregulated and downregulated expression of these microRNAs. This finding suggests that any change in microRNA expression is associated with a lower survival rate in patients with LSCC. The apparent overall heterogeneity between these studies using OS as a survival endpoint was found to be high (I^2^ = 88.05;). Similar results were found in DFS (Figs. 2, 3 and 4).

### Subgroup analysis

**Fig. 5.**
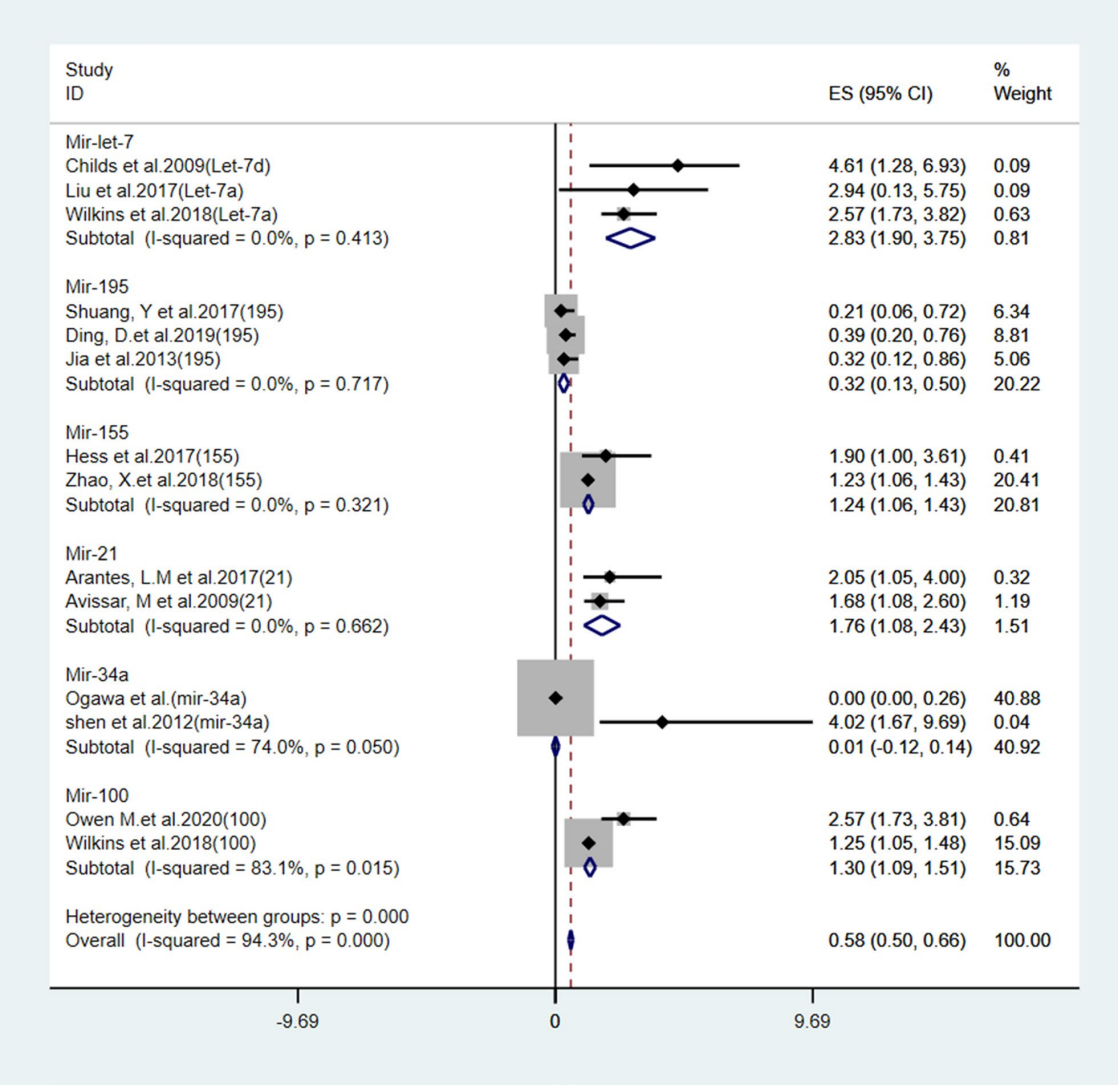
The total Forest plot of the 6 miRNA subgroups in LSCC

**Fig. 6.**
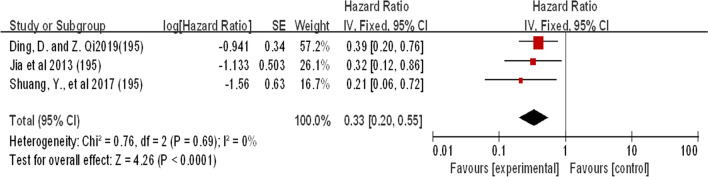
Subgroup analysis of miRNA-195 expression for OS in LSCC

**Fig. 7.**
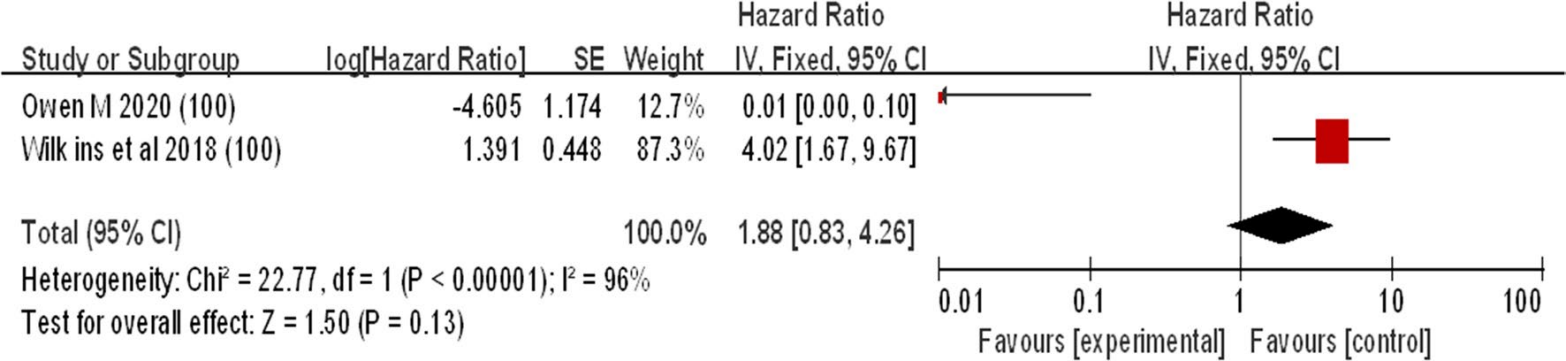
Subgroup analysis of miRNA-100 expression for OS in LSCC

**Fig. 8.**
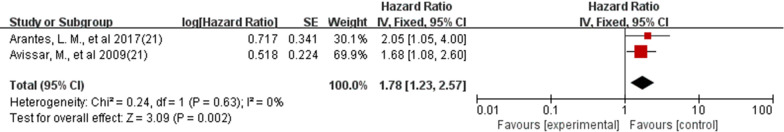
Subgroup analysis of miRNA-21 expression for OS in LSCC

**Fig. 9.**
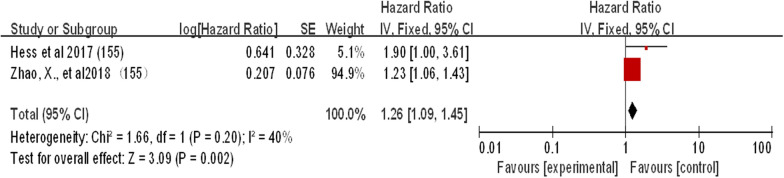
Subgroup analysis of miRNA-155 expression for OS in LSCC

**Fig. 10.**
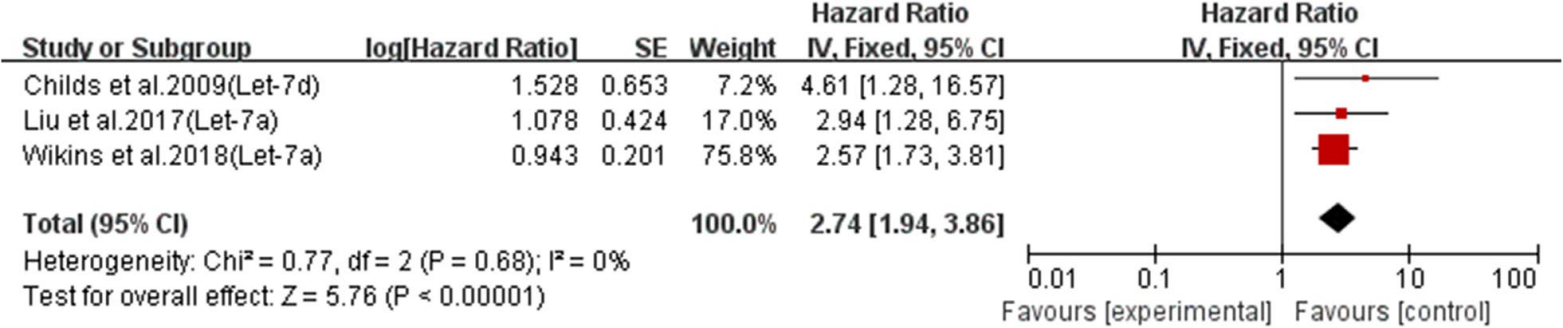
Subgroup analysis of miRNA-let-7 expression for OS in LSCC

**Fig. 11.**
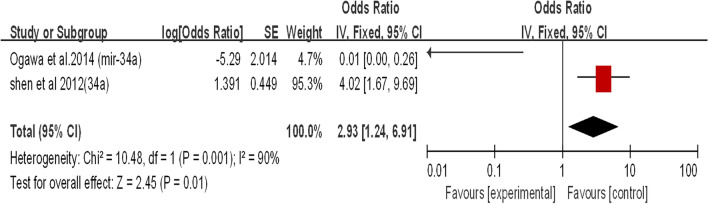
Subgroup analysis of miRNA-34a expression in LSCC (DFS)

MicroRNAs of six subgroups were included in Stata 15.1 to obtain the combined forest plot (Fig. 5). The heterogeneity between groups was large (I^2^ = 94.3%), so we conducted a subgroup analysis. We divided microRNA subgroups according to the survival end points (OS and DFS). The microRNA subgroups included miR-195, miR-100, miR-21, miR-155 and miR-let-7 for the OS group (Figs. 6, 7, 8, 9 and 10) and miR-34a for the DFS group (Fig. 11).

#### miRNA-195

Three studies revealed the expression of miR-195 in patients with laryngeal cancer (Fig. 6). All three studies showed that the expression of the miR-195 gene was downregulated in patients with LSCC. The combined effect size estimate (HR) was 0.33 (0.20–0.55; P < 0.05). All three studies showed that downregulation of miR-195 expression resulted in higher survival rates. The heterogeneity of the studies was 0 (I^2^ = 0%), indicating that the three studies supported the same finding (Fig. 6).

#### miRNA-100

Two studies investigated miR-100 expression in LSCC patients (Fig. 7). Both studies showed that miRNA-100 expression was upregulated in patients with laryngeal squamous cell carcinoma. The combined effect size estimate (HR) was 1.88 (95% CI 0.83–4.26). As shown in Fig. 7, there was significant heterogeneity between the two groups (I^2^ = 96%). The work of Owen et al. [43] contradicts the work of another author. To identify the source of the difference, we searched existing databases and found that high miR-100 expression levels were associated with lower survival rates in oral squamous cell carcinoma (46 cases) and esophageal carcinoma (47 cases). By removing this study, the merger heterogeneity effect of the upregulated group will be reduced (Fig. 7).

#### miRNA-21

Two studies reviewed the expression of miR-21 in patients with laryngeal squamous cell carcinoma (Fig. 8). The two studies demonstrated similar results regarding miR-21 expression in laryngeal squamous cell carcinoma (I^2^ = 0%). This similarity in results indicated that the two studies agreed that overexpression of miR-21 in laryngeal cancer tissue leads to poor survival. The combined effect estimate (HR) was statistically significant, with a value of 1.78 (95% CI 1.23–2.57; P < 0.05) (Fig. 8).

#### miRNA-155

Two studies reviewed miR-155 expression in patients with laryngeal squamous cell carcinoma (Fig. 9). The combined heterogeneity of the two studies was very low (I^2^ = 40%) and thus could be ignored. Both studies showed that overexpression of miR-155 in laryngeal squamous cell carcinoma resulted in reduced survival. The combined effect estimate (HR) value was 1.26 (95% CI 1.09–1.45; P < 0.05) (Fig. 9).

#### miRNA-let-7

Three studies reviewed the expression of miR-let-7 in patients with laryngeal squamous cell carcinoma (Fig. 10). The three studies demonstrated similar results regarding miR-let-7 expression in laryngeal squamous cell carcinoma (I^2^ = 0%). This similarity in results indicated that the three studies agreed that overexpression of miR-let-7 in laryngeal cancer tissue leads to poor survival. The combined effect estimate (HR) was statistically significant, with a value of 2.74 (95% CI 1.94–3.86; P < 0.05) (Fig. 10).

### Disease-free survival group

#### miRNA-34a

Two studies reviewed the expression of miR-34a in LSCC patients (Fig. 11). Both studies showed that miR-34a was downregulated in laryngeal squamous cell carcinoma. The combined effect size (HR) was 2.93 (95% CI 1.24–6.91; P < 0.05). In Fig. 11, we can clearly see that when the two studies were combined, the overall heterogeneity was high (I^2^ = 90%). Based on a review of published studies, it has been demonstrated that downregulation of miR-34a promotes laryngeal cancer cell proliferation and migration by targeting cyclin D1 [49]. The prognostic results obtained by Ogawa et al. contradicted the experimental phenomenon. Therefore, we consider that the study of Ogawa et al. led to a high degree of heterogeneity (Fig. 11).

### Publication bias

#### Funnel plot

**Fig. 12.**
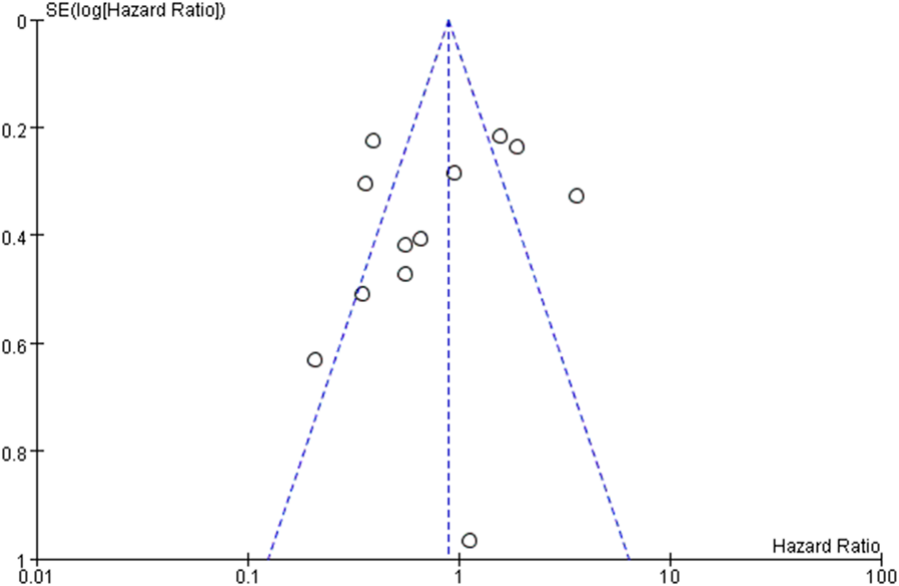
Funnel plot of studies correlating overall patient survival and downregulated miRNA expression

**Fig. 13.**
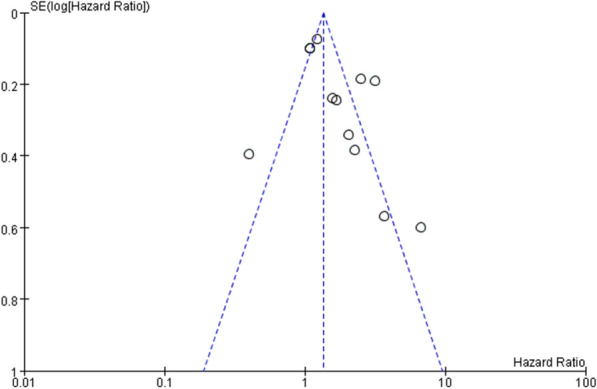
Funnel plot of studies correlating overall patient survival and upregulated miRNA expression

Figures 12 and 13 indicate that in terms of survival results, the funnel chart shows slight asymmetry between different studies. In the studies included in the analysis, visual observation of the funnel shape was the first step we used to assess publication bias. The evidence of asymmetry revealed the potential publication bias, and the publication bias of OS and DFS was detected by Begg and Egger methods. This suggests that a few studies may have been missing from our search, including results related to increased survival (Figs. 12, 13).

## Discussion

MicroRNAs are a class of nonprotein coding small RNAs 21–25 nucleotides in length which play a key role in gene regulation [50]. Circulating biomarkers are often used by clinicians to diagnose the early stages of LSCC. In contrast, invasive examination is not conducive to observing the progress of LSCC, and patients often reject invasive procedures because the examination may cause them pain. MicroRNAs are very stable, and clinical studies investigating their role have provided reproducible and consistent results [51]. In addition, we can easily detect microRNAs by RT qPCR for LSCC diagnosis. Circulating microRNAs can resist RNase A digestion and other adverse conditions, including repeated freeze-thaw cycles and long-term storage under unsuitable pH conditions [52]. MicroRNAs are promising biomarkers for early diagnosis and prognosis [53].

Our meta-analysis examined 36 articles including a patient population of 5307 individuals. In the different studies, there were 10 upregulated microRNAs and 14 downregulated microRNAs. Then, according to the expression level, they were divided into two groups, which were put into the tool to obtain the forest map. It is generally believed that if I^2^ is less than 50% this means that the heterogeneity between studies is small, and if it is greater than 50% then the heterogeneity is large. It does not make sense to combine HR of all microRNAs because their heterogeneity is too high. For prognostic biomarkers, it is more appropriate to focus on the prognostic efficiency of each microRNA.

In this study, six microRNAs (miR-34a, miR-195, miR-100, miR-21, miR-155 and miR-let-7) had high prognostic value. The remaining microRNAs were of low value. The two groups that showed great heterogeneity were miRNA-34a and miRNA-100. We tried to find the source of the heterogeneity by exploring the basic biomolecular experimental findings. We conclude that high expression of five microRNAs (miR-34a, miR-100, miR-21, miR-155 and miR-let-7) indicates poor overall survival, while high expression of miRNA-195 indicates improved overall survival. DFS is only reported only in miR-34a.

In 2008, Thian-Sze et al. conducted the first study to detect the expression level of miR-195 in head and neck tumors. Real-time quantitative PCR was used to evaluate the upregulation of miR-195 expression in four types of tongue cancer cells [54]. In 2019, Tian et al. studied two human laryngeal squamous cell carcinoma cell lines with different in vitro radiosensitivity. It was found that miR-195 is a direct downstream target of DGCR5. Moreover, the overexpression of miR-195 enhanced the radiosensitivity of Hep-2R cells [55].

Lidia et al. isolated extracellular vesicles from the plasma of 60 patients with thyroid cancer in 2020 and analyzed vesicle microRNAs. The area under the curve (AUC) value of miR-let-7 was 0.814 [56]. This result shows that miR-let-7 has a high diagnostic ability.

Yao et al. confirmed that the miR-34a-5p/Axl axis plays an aggressive role in oral cancer cells through the Akt/GSK-3 and β/β-catenin/Snail signal transduction pathways and may be a therapeutic target for oral squamous cell carcinoma [57].

The latest study by Weina et al. found that in the epithelial-mesenchymal transition of LSCC cells induced by TGF-β, miR-155HG can regulate epithelial–mesenchymal transition markers through the miR-155/SOX10 axis [58]. The author concluded that the miR-155HG/miR-155-5p/SOX10 axis is a key part of the process in promoting LSCC.

The studies in this analysis were drawn from all currently available LSCC studies that investigate microRNA expression and prognosis. Compared with other studies, the results of this study show certain advantages in accuracy. One of the shortcomings of our analysis is that the sample size of this study is relatively small. Because our study was retrospective, we were limited by the existing literature. In addition, we only chose the study endpoints of OS and DFS because of the lack of other survival outcomes, such as relapse-free survival. In addition, many risk factors contribute to the development of laryngeal squamous cell carcinoma. In the future, we can also collect data on early glottic diseases that suggest the occurrence of laryngeal cancer.

## Conclusions

Overall, despite the limitations of our review, our data still provide evidence suggesting that miRNA-100, miR-155, miR-21, miR-34a, miR-195 and miR-let-7 are potential noninvasive and simple humoral tumor markers for laryngeal cancer.

## Data Availability

All data are included in this article.

## Abbreviations

miRNA: MicroRNA
LSCC: Laryngeal squamous cell carcinoma
NA: Not available
DFS: Disease-free survival
OS: Overall survival
y: Years
N0: No lymph node metastasis
N+: Lymph node metastasis
Mean: Mean years
HNSCC: Head and neck cancer
HR: Hazard ratio
OSCC: Oral squamous cell carcinoma
RFS: Relapse free survival
DGCR5: DiGeorge syndrome critical region gene 5
TGF-β: Transforming growth factor-β
